# Evaluation of Antimicrobial and Preservative Effects of Cinnamaldehyde and Clove Oil in Catfish (*Ictalurus punctatus*) Fillets Stored at 4 °C

**DOI:** 10.3390/foods13101445

**Published:** 2024-05-08

**Authors:** Rosemary I. Ebirim, Wilbert Long

**Affiliations:** Department of Human Ecology, Delaware State University, 1200 North Dupont Highway, Dover, DE 19901, USA

**Keywords:** storage, spoilage, essential oil, cinnamaldehyde, clove oil, catfish, absorbent food pads

## Abstract

This study aimed to evaluate cinnamaldehyde (CN) and clove oil (CO) effectiveness in inhibiting growth and killing spoilage and total aerobic bacteria when overlaid with catfish fillet stored at 4 °C. A 1.00 mL concentration of CO inhibited growth by 2.90, 1.96, and 1.96 cm, respectively, for *S. baltica*, *A. hydrophilia*, and total bacteria. Similarly, treatment with 1.00 mL of CN resulted in ZIB of 2.17, 2.10, and 1.10 cm, respectively, for *S. baltica*, *A. hydrophilia*, and total bacteria from catfish exudates. Total bacteria from catfish exudates treated with 0.50 mL CN for 40 min, resulted in a 6.84 log decrease, and treatment with 1.00 mL resulted in a 5.66 log decrease at 40 min. Total bacteria exudates treated with 0.50 mL CO resulted in a 9.69 log reduction at 40 min. Total bacteria treated with 1.00 mL CO resulted in a 7.69 log decrease at 7 days, while untreated pads overlaid with catfish resulted in ≥9.00 CFU/mL. However, treated absorbent pads with catfish at 7 days, using 0.50 mL and 1.00 mL CN, had a bacterial recovery of 5.53 and 1.88 log CFU/mL, respectively. Furthermore, CO at 0.50 mL and 1.00 mL reduced the bacteria count to 5.21 and 1.53 log CFU/mL, respectively, at day 7.

## 1. Introduction

High protein content, water activity, and pH, amongst other things, limit storage, safety, and consumption, thus the need for different methods of preservation [1,2]. Fish and fish products are rich in protein, vitamins, minerals, and, often, essential omega 3 fatty acids, which have been linked to health benefits including improved longevity, promotion of fetal development, and improvement of cardiovascular function and health [3,4,5]. Fish have been associated with numerous foodborne outbreaks and foodborne illnesses [6,7]. The rate at which the human population is growing has led to an increased need for sources of protein such as fish. Fish harvesting and consumption have increased at different levels and have resulted in a global industry with increased international exports, providing employment, especially in rural riverine areas where the main occupation is fishing [8,9,10,11]. Specific spoilage organisms (SSOs) such as *Shewanella baltica* (NCTC strain 10735) and *Aeromonas hydrophila* (ATCC strain 7966) have been identified as producing metabolites that affect the sensory properties of seafood and impact the economic value chain, consumption, and utilization of fish [12,13]. Additionally, fish spoilage and contamination may make the fish unsafe and unfit for human consumption. The SSOs that affect fish are commonly associated with specific fish species, processing, storage conditions, and microbial interactions [14,15,16]. One of the most conventional preservation techniques used in the seafood industry is cold treatment. Cold processing and preservation treatments including refrigeration, chilling, super-chilling, and freezing, with or without non-synthetic or synthetic chemical preservatives, have shown effectiveness in limiting or reducing pathogens in seafood [15,17].

The use of several synthetic chemical preservatives such as nitrites, nitrates, benzoates, sulfur dioxide, and many more to destroy or delay the growth of bacteria, yeast, and molds has been applied in food preservation. These chemical preservatives have shown the ability to prevent changes in texture and color, the development of unpleasant flavors and off-odors, and the loss of nutrients in seafood during storage at low temperatures [18,19,20]. However, their use has been linked to potential hazards that can cause serious health issues, including allergic reactions, asthma, neurological damage, and cancer [19,21]. Biological methods of food preservation include the use of plant materials. Some of these plants may contain antimicrobials, antioxidants, and natural extracts that have been harnessed for food preservation [22,23,24].

Natural extracts of plant origin are gaining significant interest in the food industry as consumers demand safer and more natural preservatives. Their secondary metabolite content, especially those with phenolic groups, are considered the most effective [25,26]. They are extracted from different plant parts like flowers, bark, herbs, wood, leaves, seeds, buds, twigs, fruit, and roots. These phenolic compounds include eugenol, thymol, carvacrol, vanillin, allicin, cinnamic, aldehyde, and allyl isothiocyanate and are usually found in plants such as cloves, cinnamon, thyme, oregano, orchids, garlic, and mustard. They are being considered as preferable antimicrobial alternatives to synthetic preservatives [25,27,28]. Clove oil and cinnamaldehyde have been generally recognized as safe (GRAS) for their intended use as flavoring agents in food by the Flavor and Extract Manufacturers Association of the USA (FEMA) (21 CFR 182.60) and the Food and Drug Administration of the United States (FDA) [9,28].

Cinnamaldehyde [C6H5CH=CHCHO] is an organic aromatic aldehyde, a natural extract derived from the inner bark and leaves of cinnamon trees of the genus Cinnamomum (e.g., *C. zeylanicum*, *C. cassia*, and *C. camphor*) [26]. It is a yellow essential oil with a cinnamon odor and sweet taste. It has a wide application in the food industry, herbal remedies, and home care products. Due to its diverse applications, CN is a potential biomarker that can be used for tracing and authentication of various products [29,30]. It is commonly used in washing solutions as a decontaminant, in active food packaging as a hurdle, or incorporated in food packages as an antimicrobial agent [27,31,32]. Various studies have shown the antimicrobial activity of cinnamaldehyde against different species of microorganisms [31,33,34,35].

Clove oil has traditionally been used as a seasoning and as an antimicrobial agent in food and food packages. It has also been used as an antiseptic for oral infections. Clove oil is an essential oil derived from clove trees known as *Syzygium aromaticum* [36,37]. The main active compounds in clove oil are eugenol, eugenyl acetate, and caryophyllene. It is a yellow or colorless essential oil with a spicy, pungent taste [38,39,40]. It has a wide range of applications in the food industry and the oral hygiene industry as well. Studies have shown its antimicrobial actions against a good number of pathogenic organisms and also spoilage organisms. It has both antioxidant and scavenging activity, as reported by [41].

Catfish refers to any fish of the order *Siluriformes*, which is predominantly composed of freshwater stout-bodied and scaleless bony fish, known for their long barbels that are present near the mouth of the fish and resemble a cat’s whiskers [42]. They are bottom dwellers that mostly scavenge and feed on almost any kind of animal or vegetable matter, which exposes them to a wide range of pollutants that are toxic to humans and may result in significant levels of potential contamination [43]. Catfish are of considerable commercial and economic importance, and many of the larger species are farmed. The highly diverse nature of catfish can be seen by their wide distribution in tropical South America, Asia, and Africa; however, only one family is native to North America and one family to Europe. In the United States, the three primary species of catfish, are blue catfish, channel catfish, and flathead catfish, which are found in most rivers, lakes, and reservoirs of the United States and are readily available for food consumption [42,44,45,46].

Absorbent food pads amended with natural antimicrobial agents have been applied in the food industry as a means of actively packaging food products to reduce microbial contamination, thereby extending shelf life and promoting food safety and the sanitary conditions of refrigerated food products to improve consumer acceptability [47,48,49]. Various studies have been carried out where antimicrobial substances or materials have been incorporated into absorbent food pads [50,51,52]. For example, in one such study absorbent food pads were treated by spraying oregano essential oil of 1.5% distillate water on meat exudate absorbent pads used to extend the shelf life of overwrap packed fresh chicken drumsticks stored at 4 °C by approximately 2 days, according to a study carried out by Oral et al. [52].

When evaluating antimicrobials to be used in food packaging for shelf life capabilities, it is important to understand their ability to inhibit bacterial growth and minimal inhibitory concentration (MIC) as well as to reduce bacterial concentrations. Therefore, the purpose of this study was to investigate if essential oils of CN and CO might be used in catfish packaging as a preservative. Specifically, would CN and CO independently reduce microbial population recovery from absorbent food pads in direct contact with catfish fillets stored at 4 °C; inhibit the growth of SSOs and aerobic bacteria from catfish; and reduce the recovery of viable aerobic bacteria from catfish exudates, thereby extending the shelf life of catfish fillet in cold storage.

## 2. Materials and Methods

### 2.1. Antimicrobials Formulation

The essential oils used in this study were of commercial grade. Extracts of CN were purchased from Sigma-Aldrich (St. Louis, MO, USA). Samples were 100% pure and were extracted by steam distillation; similarly, CO was purchased from Piping Rock Health Products LLC (Ronkonkoma, NY, USA) and was 100% pure and extracted by steam distillation. The preparation of the essential oil treatment concentrations was carried out by diluting and mixing tap water in a laboratory vortex at room temperature directly before use.

### 2.2. Bacterial Strains, Growth, Inoculum Preparation and Zone of Inhibition

Specific spoilage organisms (SSOs), *Shewanella baltica* (NCTC strain 10735), and *Aeromonas hydrophila* (ATCC strain 7966), stored at −80 °C were obtained from the Food Biotechnology Laboratory in the Department of Human Ecology, College of Agriculture, Science, and Technology at Delaware State University. A loop of the SSO stock cultures was inoculated into separate 10 mL tryptic soy broth (TSB; Carolina Biological Supply Co., Burlington, NC, USA) and allowed to grow for 24 h at 27 and 37 °C, respectively. Thereafter, 1.00 mL was taken and plated on tryptic soy agar (TSA; MP Biomedicals, LLC (Solon, OH, USA). After that, treatment concentrations using 5 mL of tap water with concentrations of 0.125, 0.25, 0.50, 0.75, and 1.00 (mL/mL) CN or CO were prepared independently. Then, 20 µL of each treatment concentration was spot inoculated independently on the TSA plates containing the SSOs to determine the zone of inhibition. Commercial catfish were purchased from seafood markets within Dover, DE, USA. Samples were aseptically dived into 5 g pieces, which was stomached using 5.00 mL of TSB for 30 s. Then, 1.00 mL of the exudates was plated onto TSA. Twenty microliters of the essential oil treatment concentrations were spot inoculated on each TSA plate containing exudates from the catfish fish samples and incubated at an optimal growth temperature of 27 °C for 24 h to check for the zone of inhibition.

### 2.3. Bacterial Death Curve 

Five grams of catfish fillet sample was stomached using 5 mL TSB, and 1 mL of stomached exudate from catfish fillets was plated on TSA and incubated for 24 h at 27 °C. Afterwards, loops of bacteria colonies isolated from the incubated stomached exudates were inoculated in 10 mL TSB tubes for use for each of the different treatment concentrations and incubated for 24 h at 27 °C. Thereafter, 20 mL of the incubated samples were mixed homogeneously in 50 mL conical tubes for each of the different treatment concentrations. The essential oil treatment concentrations of 0.50 and 1.00 mL CN or CO were then added to the 20 mL sample tubes at a 1:10 mL concentration and vortexed for 5 s. At 5, 10, 20, 30, and 40 min, 1.00 mL of the sample mixtures was pipetted and serially diluted in TSB. Then, 1.00 mL was removed from serially diluted tubes and plated on TSA. The plates were incubated for 24 h at 27 °C and bacterial death was enumerated.

### 2.4. Absorbent Food Pad Inoculation

Absorbent food pads were inoculated using a modified technique by Ren et al. [53]. Dri-loc absorbent food pads were donated by Novipax (Oak Brook, IL, USA). Treatment of the absorbent food pads and packaging materials was conducted by immersing the absorbent food pads into essential oil treatment concentrations of 0.50 and 1.00 mL of CN or CO, respectively, at a 1:10 ratio (mL/mL) and then allowing them to sit for 30 min at room temperature. Approximately, 50 g of untreated catfish fillets were aseptically cut with a sterilized knife and placed on both treated and untreated absorbent food pads and then stored for 7 days at 4 °C. The effect of the treatments over time was evaluated on days 1, 3, 5, and 7, respectively, by stomaching each treated absorbent food pad and each untreated control absorbent food pad in 5.00 mL of TSB. One milliliter was taken from each stomached absorbent food pad sample and then serially diluted in TSB tubes, and 1.00 mL was plated on TSA to enumerate the total aerobic bacteria/mL on the absorbent food pads.

### 2.5. Statistical Analysis

Using the SPSS statistical software program 26 (SPSS Inc., Chicago, IL, USA), one-way analysis of variance (ANOVA) was applied in this study. Tukey’s HSD test was used for the comparison of logarithmic values to determine statistical significance between mean values of various treatments. Standard deviation was calculated for the data generated using Microsoft Excel 2016 (Microsoft Inc., Redmond, WA, USA). All experiments were carried out in triplicate. Significance was defined as *p* ≤ 0.05.

## 3. Results

### 3.1. Zone of Inhibition

The inhibitory effects of different concentrations (0.125, 0.25, 0.50, 0.75, and 1.00 mL) of CN or CO are seen in Table 1. Cinnamaldehyde exhibited ZIB ranging from 1.00 to 2.10 cm. At 0.125 mL, CN to *A. hydrophila* plates exhibited a ZIB of 1.00 ± 0.00 cm. The zone of inhibition significantly increased to 1.40 ± 0.07 cm when the treatment concentration increased to 0.25 mL. At a concentration of 0.50 mL CN, the ZIB significantly increased even further to 1.70 ± 0.01 cm from the previous CN concentration. However, there was no significant difference (*p* ≤ 0.05) in ZIB observed on *A. hydrophila* plates when treated with 0.50 and 0.75 mL CN. At 1.00 mL, CN treatment further increased the ZIB to 2.10 ± 0.17 cm, with no significant difference from the previous concentration. Furthermore, *A. hydrophila* plates treated with 0.125, 0.25, 0.50, 0.75, or 1.00 mL CO exhibited ZIB ranging from 1.03–1.96 cm. At all levels, with increasing concentrations of CO treatment ZIB increased significantly. At 0.125 mL CO, *A. hydrophila* treated plates revealed a ZIB of 1.03 ± 0.05 cm, which slightly increased to 1.23 ± 0.11 cm when treated with 0.25 mL CO. Upon increasing the concentration to 0.50 mL CO, the ZIB increased to 1.50 ± 0.00 cm with significance difference from the two previous treatment concentrations. Upon further increase of CO concentration to 0.75 mL, there was a further increment in the ZIB to 1.90 ± 0.17 cm. Further increasing the concentration of CO to 1.00 mL resulted in a maximum ZIB of 0.46 cm in diameter.

Both CN and CO revealed the ability to prevent *A. hydrophila* growth. However, CN and CO treatments at 0.75 mL and 1.00 mL concentrations had no significant difference (*p* > 0.05) in their bacteriostatic effects on *A. hydrophila*. Table 2 shows *Shewanella baltica* plates treated with different concentrations of CN or CO (0.125, 0.25, 0.50, 0.75, and 1.00 mL), where CN showed ZIB ranging from 1.03 to 2.17 cm. At 0.125 mL concentration of CN, a ZIB of 1.03 ± 0.06 cm was observed. No significant difference (*p* ≤ 0.05) was observed when CN concentration increased to 0.25 mL. When the concentration of CN was further increased to 0.05 mL, a ZIB of 1.90 ± 0.01 cm resulted. No further significant increase (*p* > 0.05) was observed from 0.50 to 1.00 mL.

Clove oil treatment concentrations on *S. baltica* inoculated plates resulted in ZIB ranging from 0.80 ± 0.00 to 2.95 ± 0.07 cm in diameter. Control, untreated inoculated plates resulted in no ZIB. At 0.125 mL concentration of CO in *S. baltica*, a ZIB of 0.80 ± 0.00 cm was seen. This value was not statistically different (*p* > 0.05) with 0.125 mL and 0.25 mL of CN treatment of *S. baltica.* The ZIB was increased significantly (*p* ≤ 0.05) by 0.95 cm when the concentration of CO was increased to 0.25 mL. Increasing the concentration to 0.50 mL, 0.75 mL, and 1.00 mL did not result in any additional significant difference (*p* > 0.05) in the ZIB.

The inhibitory effects of CN or CO on catfish total aerobic bacteria are shown in Table 3. Catfish plates treated with 0.125, 0.25, 0.50, 0.75, and 1.00 mL of CN exhibited ZIB ranging from 0.80 to 1.10 cm. At 0.125 mL CN treatment, catfish plates exhibited a ZIB of 0.80 ± 0.00 cm, and no significant difference (*p* > 0.05) was observed when CN concentration increased up to1.00 mL.

Catfish plates treated with 0.125, 0.25, 0.50, 0.75, and 1.00 mL concentrations of CO exhibited ZIB ranging from 1.03 ± 0.05 to 1.96 ± 0.05 cm. At 0.125 mL CO, catfish treated plates revealed a ZIB of 1.03 ± 0.05 cm. No significant difference (*p* > 0.05) was observed with 0.25 mL CO treatment of catfish plates. Upon increasing the concentration to 0.50 mL CO, the ZIB increased to 1.50 ± 0.00 cm. Further increase of CO concentration to 0.75 mL and 1.00 mL, there was a significant (*p* ≤ 0.05) increase in the ZIB from the previous CO concentrations 1.90 ± 0.17cm and. 1.96 ± 0.05 cm, respectively. However, there was no significant difference (*p* > 0.05) between both treatments.

### 3.2. Bacterial Death Curve

**Figure 1:**
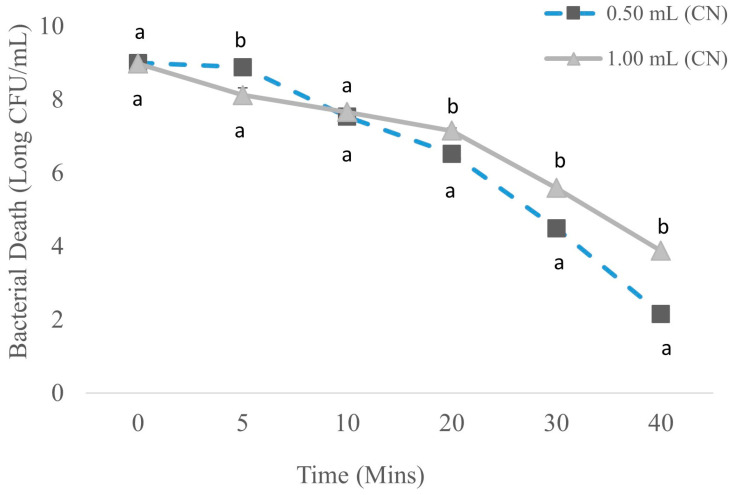
Reduction of bacterial count in store-bought catfish treated with CN. n = 3 for each concentration on each day. Concentration of treatment to water was at 1:10 mL per each treatment. Significance was defined as *p* ≤ 0.05. Differences within each day are represented by a and b. Data were analyzed by one-way ANOVA.

Figure 1 shows that catfish exudates in TSB at time zero and treated with 0.50 mL and 1.00 mL CN had 9.00 ± 0.06 and 8.98 ± 0.03 log CFU/mL, respectively. At 5 min after 0.50 mL and 1.00 mL addition, bacterial concentrations for 0.50 mL did not significantly (*p* > 0.05) decrease. However, for 1.00 mL bacterial counts decreased on average by 0.86 log to 8.12 log CFU/mL. When increasing the length of treatment to 10 min, bacterial concentrations for 0.50 mL and 1.00 mL decreased on average by 1.35 log to 7.53 ± 0.01 log CFU/mL and 0.46 log to 7.66 ± 0.01 log CFU/mL, respectively. Bacterial concentration was further significantly (*p* ≤ 0.05) reduced at 20 min for both treatment concentrations, on average by 1.01 log to 6.52 ± 0.01 log CFU/mL and 0.50 log and 7.15 ± 0.06 log CFU/mL, respectively. Thirty minutes after treatment with 0.50 mL of CN, bacterial counts decreased on average by 2.03 log and dropped to 4.49 ± 0.03 log CFU/mL, and for 1.00 mL concentration, the bacterial concentration decreased by 1.56 log and dropped to 5.59 ± 0.11 log CFU/mL. At 40 min after treatment, 0.50 mL bacterial counts significantly (*p* ≤ 0.05) reduced from the previous concentration by 2.33 log to 2.16 ± 0.08 log CFU/mL. Similarly, at 40 min 1.00 mL CN reduced the average bacterial counts by 1.71 log to 3.88 ± 0.03 log CFU/mL.

**Figure 2:**
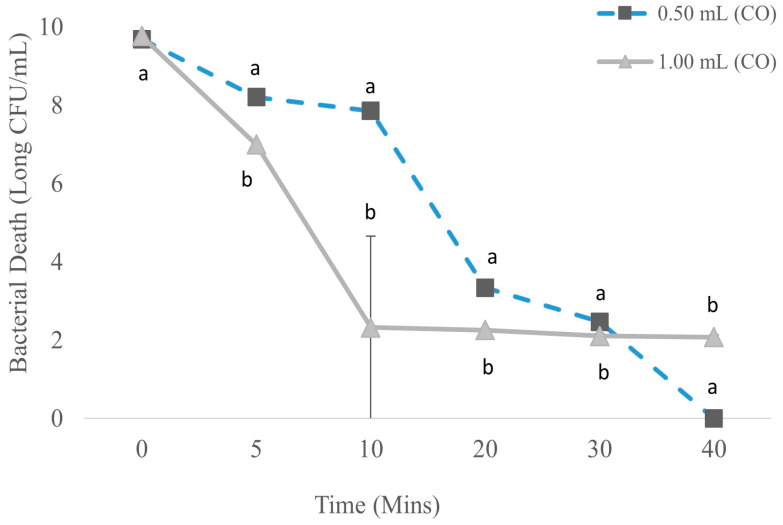
Reduction of bacterial count in store-bought catfish treated with CO. n = 3 for each concentration on each day. Concentration of treatment to water was at 1:10 mL per each treatment. Significance was defined as *p* ≤ 0.05. Differences within each day are represented by a and b. Data were analyzed by one-way ANOVA.

Figure 2 shows the bactericidal effects of CO on catfish exudates in TSB. At time zero min, when treated with 0.50 and 1.00 mL CO the catfish exudates had 9.69 ± 0.02 and 9.77 ± 0.03 log CFU/mL, respectively. At 5 min after 0.50 and 1.00 mL addition of CO, bacterial concentrations significantly (*p* ≤ 0.05) decreased, on average by 1.48 log to 8.21 ± 0.06 and 2.77 to 7.00 ± 00 log CFU/mL, respectively. When increasing the treatment time to 10 min, the bacterial counts for 0.50 mL further decreased significantly (*p* ≤ 0.05) by 0.35 log to 7.86 ± 0.15 and 4.67 to 2.33 ± 10 log CFU/mL. However, there was no significant (*p* > 0.05) decrease in bacterial concentration for CO 1.00 mL from 20 up to 40 min. At 0.50 mL concentrations at 20 and 30 min, the bacterial concentration further significantly (*p* ≤ 0.05) reduced, on average by 4.52 log to 3.34 ± 0.02, and 0.87 log to 2.47 ± 0.05 and 0.32 log, respectively. When further increasing the time of treatment to 40 min for 0.50 mL concentrations, there were no counts of viable bacteria at the lowest recovery level.

**Figure 3:**
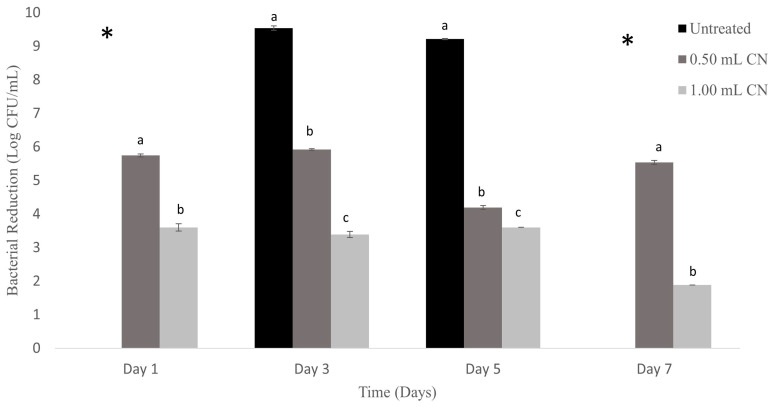
Absorbent food pads treated with CN for storage of catfish at 4 °C. * represents plates that were above the highest level of bacteria count, too numerous to count. n = 3 for each concentration and trial. One mL of each prepared concentration was added to 5 mL of bacteria. The different letters (a, b, c) indicate significant differences between treatments on each day. Significance was defined as *p* ≤ 0.05. Statistical analysis for this study was conducted by means of one-way ANOVAs.

In Figure 3, untreated absorbent food pads from catfish packaging stayed at ≥9 log CFU/mL for 7 days of study. Absorbent food pads treated with 0.50 mL CN resulted in observably lower bacterial counts on days 1 3, 5, and 7, with results of 5.74 ± 0.04 log CFU/mL, 5.92 ± 0.02 log CFU/mL, 4.19 ± 0.05 log CFU/mL, and 5.53 ± 0.03 log CFU/mL, respectively. Of these treatments, the lowest significant (*p* ≤ 0.05) reduction was observed on day 5. Absorbent pads treated with 1.00mL on days 1, 3, 5, and 7, showed results of 3.59 ± 0.011 log CFU/mL, 3.39 ± 0.02 log CFU/mL, 3.60 ± 0.01 log CFU/mL, and 1.31 ± 0.11 log CFU/mL, respectively. Observable reductions were seen each day between CN treatments of 0.50 mL and 1.00 mL of 2.51, 2.53, 0.59, 4.22, and 2.6 log CFU/mL, respectively.

Our results suggest that the application of CN to absorbent food pads overlaid with catfish fillet can reduce bacteria in absorbent food pads over 7 days of storage at 4 °C. Increasing the concentration of CN from 0.50 mL to 1.00 mL also increased the reduction of bacteria concentration in the absorbent pads.

**Figure 4:**
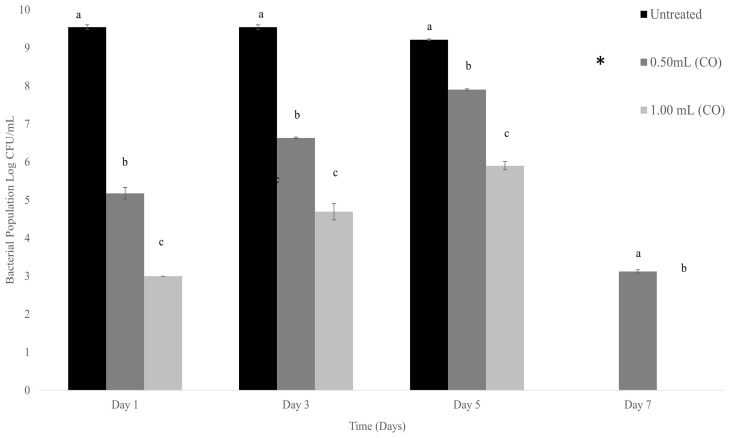
Absorbent food pads treated with CO for storage of catfish at 4 °C. * represents plates that were above the highest level of bacteria count, too numerous to count. Treatment with 1.00 mL CO resulted in no growth. n = 3 for each concentration and trial. One ml of each prepared concentration was added to 5 mL of bacteria. The different letters (a, b, c) indicate significant differences between treatments on each day. Significance was defined as *p* ≤ 0.05. Statistical analysis for this study was conducted by means of one-way ANOVAs.

We found that, as shown in Figure 4, untreated absorbent food pads with packaged catfish fillet had ≥9.59 log CFU/mL of total aerobic bacteria for all seven days. Absorbent food pads treated with 0.5 mL CO, resulted in observably lower bacterial counts on days 1, 3, 5, and 7 with results of 5.17 ± 0.15 log CFU/mL, 6.63 ± 0.02 log CFU/mL, 7.90 ± 0.04 log CFU/mL, and 3.12 ± 0.17 log CFU/mL respectively. Of these treatments, the lowest significant (*p* ≤ 0.05) reduction was observed on day 7. Absorbent pads treated with 1.00 mL on days 1, 3, 5, and 7 resulted in a bacterial reduction of 3.00 ± 0.00 log CFU/mL, 5.30 ± 0.24 log CFU/mL, 5.90 ± 0.11 log CFU/mL, respectively, and zero counts of total bacteria on day 7. Results suggest that CO embedment on absorbent food pads for storage of catfish can significantly (*p* ≤ 0.05) reduce bacteria counts in absorbent food pads over 7 days of storage at 4 °C.

In addition, sensory observations were made on some quality attributes of the catfish sample. The sensory observation and quality attributes of color, aroma, and texture were observed on days 1, 3, 5, and 7 of storage. Catfish fillet samples with untreated and treated absorbent food pads stored at 4 °C were evaluated for texture, smell, and color. Fish fillet samples with the untreated absorbent food pads had a more off-odor and slimy texture by the end of the 7 days at 4 °C of storage. Catfish samples on treated absorbent food pads had less visual degradation, especially in locations in which the fish was in direct contact with the pad. Fish fillet samples on absorbent food pads with CN had a slightly yellow color and CN aroma, while CO treated absorbent food pads had a pale-yellow color and CO aroma. The color change may potentially be due to the uptake of coloration from the natural antimicrobial preservatives CN and CO.

## 4. Discussions

Natural extracts from plant origins have been in use as antimicrobials for food safety and preservation. Various studies have shown CN and CO to be effective against some pathogenic and food spoilage organisms. Our results establish that different concentrations of CN and CO at different levels of investigation show antimicrobial effectiveness against SSOs and total aerobic bacteria from catfish. The results of this study are in line with studies carried out by [54], which used cinnamaldehyde-incorporated and eugenol-incorporated methylcellulose films as antimicrobial packaging materials to investigate antimicrobial activity against target microorganisms using both an agar-disc diffusion technique and a vapor diffusion technique. At a concentration of 50 µL/mL, cinnamaldehyde and eugenol revealed antimicrobial activity against *Aeromonas hydrophila*, *Enterococcus faecalis*, and some other test strains where ZIB ranged from 0.87 to 3.01 cm, which is in the range of the results from this study, where CN and CO showed ZIB against *Aeromonas hydrophila* ranging from 1.0 to 2.10 cm and from 1.17 to 2.40 cm, respectively. Furthermore, the average range for the ZIB of CN and CO from this study against *Shewanella baltica* was 0.87 to 2.17 cm, which is in line with the findings from [54] against some SSOs. Abdel et al. [55] reported the antimicrobial and inhibitory characteristics of clove oil seen against eight microorganisms, with CO showing a ZIB range of 2.5 and 3.6 cm at low and high concentrations against *Escherichia coli*, which is also within the range of the results obtained from this study, where CO showed a ZIB range against total aerobic bacteria from catfish of 1.03 and 1.96 cm at low and high concentrations. The broad spectrum of inhibitory effects against microorganisms by cinnamaldehyde and eugenol has been reviewed, and their activity and highlighted potential use in antimicrobial packaging agents have been reported [30,34]. This is in line with the findings of this study that showed the antimicrobial activity of CN and CO used in active packaging and absorbent food pads against total aerobic bacteria from catfish exudates.

At the highest concentrations of CN and CO used to investigate the bacteriostatic effects of the treatments, CO exhibited a larger ZIB on the SSOs and the total aerobic bacteria from catfish. Clove oil, furthermore, showed a greater bactericidal effect on the total aerobic bacteria from catfish exudates. The findings from this study also, show that treating absorbent food pads with either 0.50 mL or 1.00 mL concentrations of CN or CO, could limit the increase in bacterial count on absorbent pads used for packaging and storage of catfish fillet at 4 °C and thus extend the shelf life of fish. Furthermore, catfish fillet samples overlaid on the absorbent food pads had the aroma of either CN or CO, with slight yellowish discoloration.

## 5. Conclusions

Our results establish that the present study has revealed the effectiveness of CN and CO for controlling microbial populations for channel catfish (*Ictalurus punctatus*) fillet packaging using absorbent food pads. These natural extracts used at different concentrations and different phases in this study showed both bactericidal and bacteriostatic abilities in controlling the microbial population of specific spoilage organisms and total aerobic bacteria in absorbent food pad packaging of catfish fillet. Therefore, incorporation of CN and CO into absorbent food pads may be applied successfully in fish packaging to prevent and control bacterial spoilage of fish, especially in developing countries where there is poor infrastructural development in the area of post-harvest handling of fish to delay and reduce microbial proliferation in harvested fish before proper handling and storage. These findings indicate that natural extracts such as CN and CO can potentially extend the shelf life of fish such as catfish in effective active packaging using absorbent food pads. Some sensory characteristics of the catfish were altered in terms of color and aroma, which can be attributed to the color and aroma of the CN and CO extracts used. Therefore, their effect on sensory quality and acceptability by consumers needs to be further studied.

## Figures and Tables

**Table 1:** Zone of Inhibition of SSO (*Aeromonas hydrophila*) treated with CN and CO in sterilized tap water (mL/mL).

**Treatment Conc. (mL/mL)**	**ATCC *Aeromonas hydrophila* and CN**			
	**Trial 1 (cm)**	**Trial 2 (cm)**	**Trial 3 (cm)**	**Average**
0.125	1.00	1.00	1.00	1.00 ± 0.00 ^a^
0.25	1.40	1.50	1.30	1.40 ± 0.10 ^c^
0.50	1.70	1.80	1.60	1.70 ± 0.01 ^d^
0.75	1.90	1.90	1.80	1.85 ± 0.07 ^de^
1.00	2.00	2.30	2.00	2.10 ± 0.17 ^e^
	**ATCC *Aeromonas hydrophila* and CO**			
	**Trial 1 (cm)**	**Trial 2 (cm)**	**Trial 3 (cm)**	**Average**
0.125	1.00	1.10	1.00	1.03 ± 0.05 ^a^
0.25	1.10	1.30	1.30	1.23 ± 0.11 ^b^
0.50	1.50	1.50	1.50	1.50 ± 0.00 ^c^
0.75	1.70	2.00	2.00	1.90 ± 0.17 ^e^
1.00	2.00	2.00	1.90	1.96 ± 0.05 ^e^

Means of averages are expressed in cm ± standard deviation. Different letters (a, b, c, d, and e) superscripted on the mean indicate a significant difference in the two treatments (CN and CO). Significance was defined as *p* ≤ 0.05. Data were analyzed by one-way ANOVA.

**Table 2:** Zone of Inhibition of SSO (*Shewanella baltica*) treated with CN and CO in sterilized tap water (mL/mL).

**Treatment Conc. (mL/mL)**	**NTCC 10735, *Shewanella baltica* and CN**			
	**Trial 1 (cm)**	**Trial 2 (cm)**	**Trial 3 (cm)**	**Average**
0.125	1.00	1.10	1.00	1.03 ± 0.06 ^a^
0.25	1.20	1.10	1.00	1.10 ± 0.10 ^a^
0.50	2.00	1.90	1.80	1.90 ± 0.01 ^b^
0.75	2.20	2.00	2.00	2.07 ± 0.12 ^b^
1.00	2.30	2.20	2.00	2.17 ± 0.15 ^b^
	**NTCC 10735 *Shewanella baltica* and CO**			
	**Trial 1 (cm)**	**Trial 2 (cm)**	**Trial 3 (cm)**	**Average**
0.125	0.80	0.80	0.80	0.80 ± 0.00 ^a^
0.25	1.80	1.70	1.75	1.75 ± 0.07 ^b^
0.50	2.50	2.70	2.60	2.60 ± 0.14 ^c^
0.75	2.80	2.60	2.70	2.70 ± 0.14 ^c^
1.00	2.90	3.00	2.95	2.90 ± 0.07 ^c^

Means of averages are expressed in cm ± standard deviation. Different letters (a, b, and c) superscripted on the mean indicate a significant difference between the two treatments (CN and CO). Significance was defined as *p* ≤ 0.05. Data were analyzed by one-way ANOVA.

**Table 3:** Zone of inhibition of catfish total aerobic bacteria exudate treated with CN and CO in sterilized tap water (mL/mL).

**Treatment Conc. (mL/mL)**	**Catfish and CN**			
	**Trial 1 (cm)**	**Trial 2 (cm)**	**Trial 3 (cm)**	**Average**
0.125	0.80	0.80	0.80	0.80 ± 0.00 ^a^
0.25	0.90	0.90	0.90	0.90 ± 0.00 ^ab^
0.50	1.00	1.00	1.00	1.00 ± 0.00 ^ab^
0.75	1.00	1.00	1.10	1.03 ± 0.05 ^bc^
1.00	1.10	1.20	1.00	1.10 ± 0.10 ^bc^
	**Catfish and CO**			
	**Trial 1 (cm)**	**Trial 2 (cm)**	**Trial 3 (cm)**	**Average**
0.125	1.00	1.10	1.00	1.03 ± 0.05 ^bc^
0.25	1.10	1.30	1.30	1.23 ± 0.11 ^c^
0.50	1.50	1.50	1.50	1.50 ± 0.00 ^d^
0.75	1.70	2.00	2.00	1.90 ± 0.17 ^e^
1.00	2.00	2.00	1.90	1.96 ± 0.05 ^e^

Means of averages are expressed in cm ± standard deviation. Different letters (a, b, c, d, and e) superscripted on the mean indicate a significant difference between the two treatments (CN and CO). Significance was defined as *p* ≤ 0.05. Data were analyzed by one-way ANOVA.

## Data Availability

The original contributions presented in the study are included in the article, further inquiries can be directed to the corresponding author.
